# Supplementary material for: Orientations and water dynamics of photoinduced secondary charge-separated states for magnetoreception by cryptochrome
Source: Commun Chem. 2021 Sep 30;4:141. doi: 10.1038/s42004-021-00573-4 (PMC9814139; doi:10.1038/s42004-021-00573-4)
Supplement: Supplementary file 1 — Supplementary Information [file 42004_2021_573_MOESM1_ESM.pdf]

Supplementary Information

Orientations and Water Dynamics of Photoinduced Secondary Charge-Separated States for Magnetoreception by Cryptochrome

Misato Hamada, [1] *Tatsuya Iwata,** [2] *Masaaki Fuki,* [1,3] *Hideki Kandori,*¶ [4,5]

Stefan Weber * [6] *and Yasuhiro Kobori** [1,3]

[1] Department of Chemistry, Graduate School of Science, Kobe University, 1–1 Rokkodai–cho, Nada–ku, Kobe 657–8501, Japan

[2] Department of Pharmaceutical Sciences, Toho University, Funabashi, Chiba 274–8510, Japan

[3] Molecular Photoscience Research Center, Kobe University, 1–1 Rokkodai–cho, Nada–ku, Kobe 657–8501, Japan

[4] Department of Life Science and Applied Chemistry, Nagoya Institute of Technology, Showa-ku, Nagoya 466-8555, Japan

[5] OptoBioTechnology Research Center, Nagoya Institute of Technology, Showa-ku, Nagoya 466-8555, Japan

[6] Institute of Physical Chemistry, Albert-Ludwigs-Universität Freiburg, 79104 Freiburg, Germany

***Corresponding Authors:** Tatsuya Iwata (tatsuya.iwata@phar.toho-u.ac.jp), Stefan Weber (Stefan.Weber@physchem.uni-freiburg.de), Yasuhiro Kobori (ykobori@kitty.kobe-u.ac.jp)

Supplementary Figures

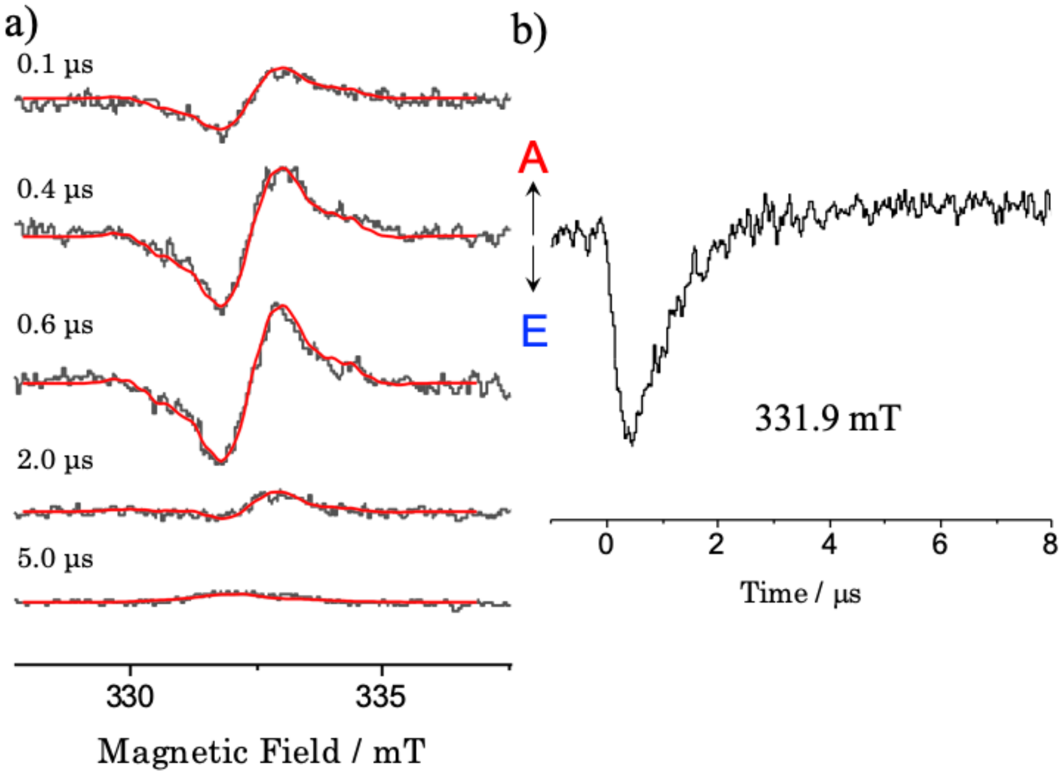

Supplementary Figure 1. a): Delay time dependence of the TREPR spectrum obtained by the depolarized 450 nm laser irradiation of *XlCry*-DASH at 240 K, showing the SCRP of the ternary CS state of $FAD^{-\bullet}\cdots W_A(H)\cdots W_B(H)\cdots W_C(H)^{+\bullet}$ For this experiment, the wild type *XlCry*-DASH solution was inserted in a sealed glass tube with the inside diameter of 0.6 mm. From the ground state absorption spectrum, the optical density from FAD was 2.0 at 450 nm light for the solution in a 10 mm cuvette. Red solid lines are calculated delay time dependence of the TREPR spectra by using the singlet-precursor SCRP model taking into account the spin-lattice relaxations (Supplementary Table 5) in the ternary CS state using the molecular geometries of FAD and $W_C(H)$ shown in Supplementary Figure 2. b) Decay curve of the TREPR signal representing long-lived RP at 240 K. The decay of the emissive polarization at 331.9 mT is due to the spin-lattice relaxations with T_1 = 1.4 μs and T_{23} = 1.8 μs in Supplementary Table 5.

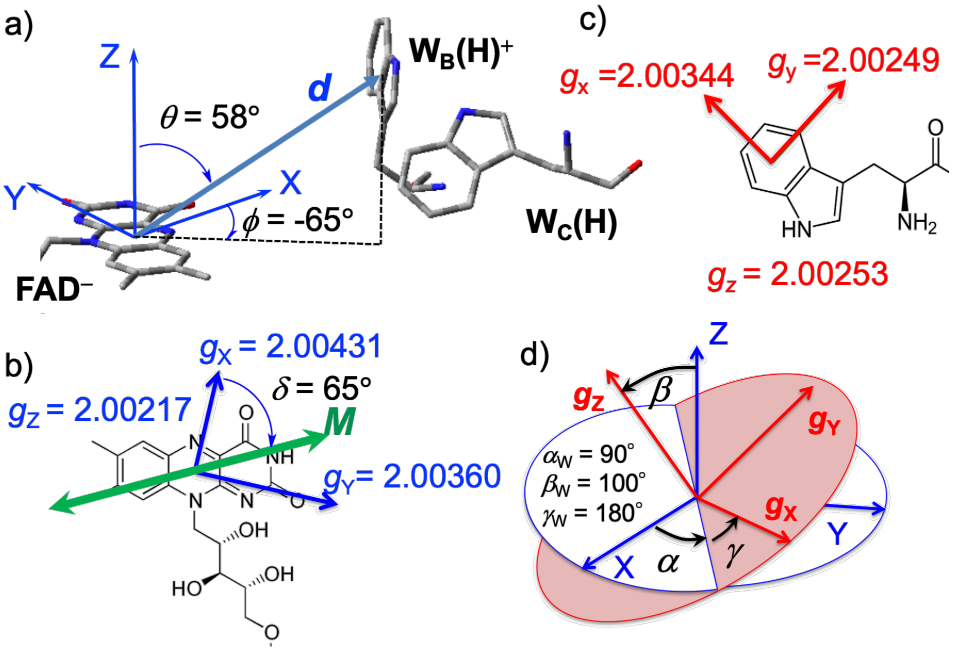

Supplementary Figure 2. Geometry Settings of the Secondary and Terminal CS states. a) Setting of the direction of the principal axis in the dipolar coupling (***d***) with applying the point-dipole approximation. The reference axes of (X, Y, Z) were taken from the principal axes of the g-tensor of flavin neutral radical because the spin density distribution of $FAD^{-•}$ is similar to that for the flavin neutral radical.[1] b) Principal axes of g-tensor (g_X, g_Y, g_Z) in $FAD^{-•}$. The principal values were taken from ref.([2]). The hyperfine parameters in $FAD^{-•}$ are listed in Supplementary Table 3. ***M*** direction of δ = 65° was set with respect to the reference axes as shown in Fig. 3a. c) Principal axes of g-tensor (g_x, g_y, g_z) in $W(H)^{+•}$. The principal values were taken from ref.([3]) in $W(H)^{+•}$. The anisotropic hyperfine parameters in $W(H)^{+•}$ are listed in Supplementary Table 4. d) Euler angles settings for charge conformations in $FAD^{-•}$ and $W_B(H)^{+•}$ at 120 K. In $FAD^{-•}$, $\alpha = \beta = \gamma = 0°$ was employed, while $\alpha_W = 90°$, β_W = 100° and γ_W = 180° were set in $W(H)^{+•}$ at 120 K. On the ternary CS state of $FAD^{-•} \cdots W_C(H)^{+•}$ to explain the delay time dependence of the TREPR spectrum in Supplementary Figure 1 at 240 K, $\alpha = \beta = \gamma = 0°$, $\alpha_W = 0°$, β_W = 30°, γ_W = 210° were employed with $\theta = 75°$ and $\phi = -80°$. These geometry parameters well coincide with the x-ray structure of FAD and $W_C(H)$ in a) from *ChlaCRY* (PDB code:5ZM0), suggesting that nuclear displacements by the stepwise charge-separations are minor in the reduced and oxidized chromophores of $FAD^{-•} \cdots W_B(H)^{+•}$ and $FAD^{-•} \cdots W_C(H)^{+•}$.

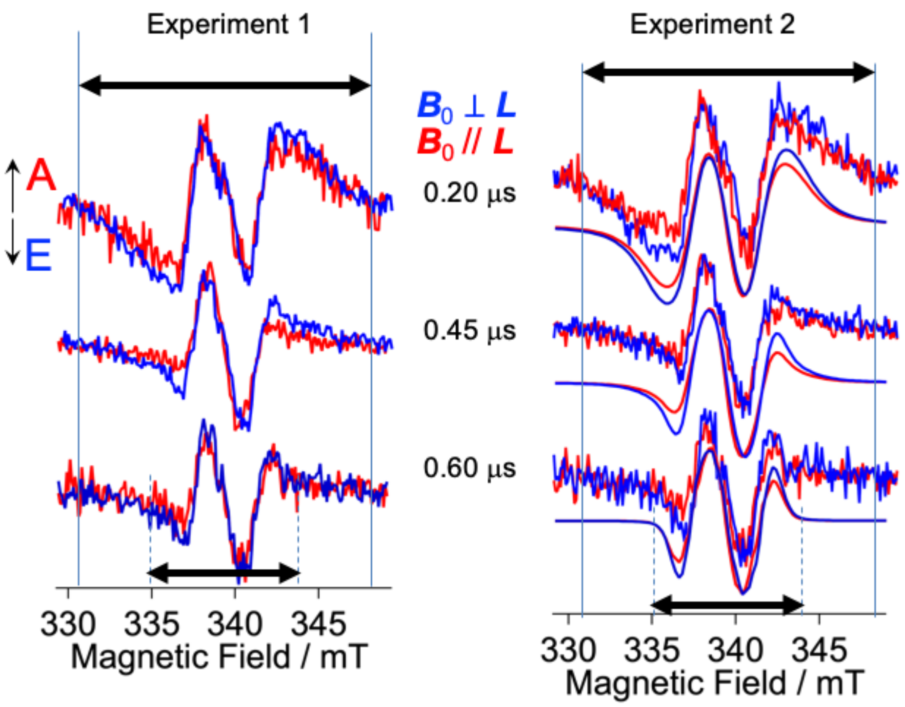

Supplementary Figure 3. Two experimental results of the magnetophotoselection measurements of WT XlCRY-DASH at 120 K by the 450 nm laser irradiations. The small but existing differences in the TREPR spectra by the direction of the laser polarization were confirmed by the additional TREPR observations (Experiment 2), showing that the outer E/A polarization is stronger in the blue spectra ($\boldsymbol{B}_0 \perp \boldsymbol{L}$ corresponding to the dashed lines in Fig. 3c) than that in the red ones for $\boldsymbol{B}_0$ // $\boldsymbol{L}$ corresponding to the solid lines in Fig. 3c. It is also evident that the entire spectrum widths are significantly larger at 0.2 μs than the widths at 0.6 μs from the black bold arrows. At the delay time of 0.2 μs, the combination of (J, T_{2J}*) = (1.5 mJ, 3 ns) only reproduced the experimental results when the x-ray conformations of FAD and W_B(H) are adopted as the reduced and oxidized species, as detailed in Supplementary Figures 4-6.

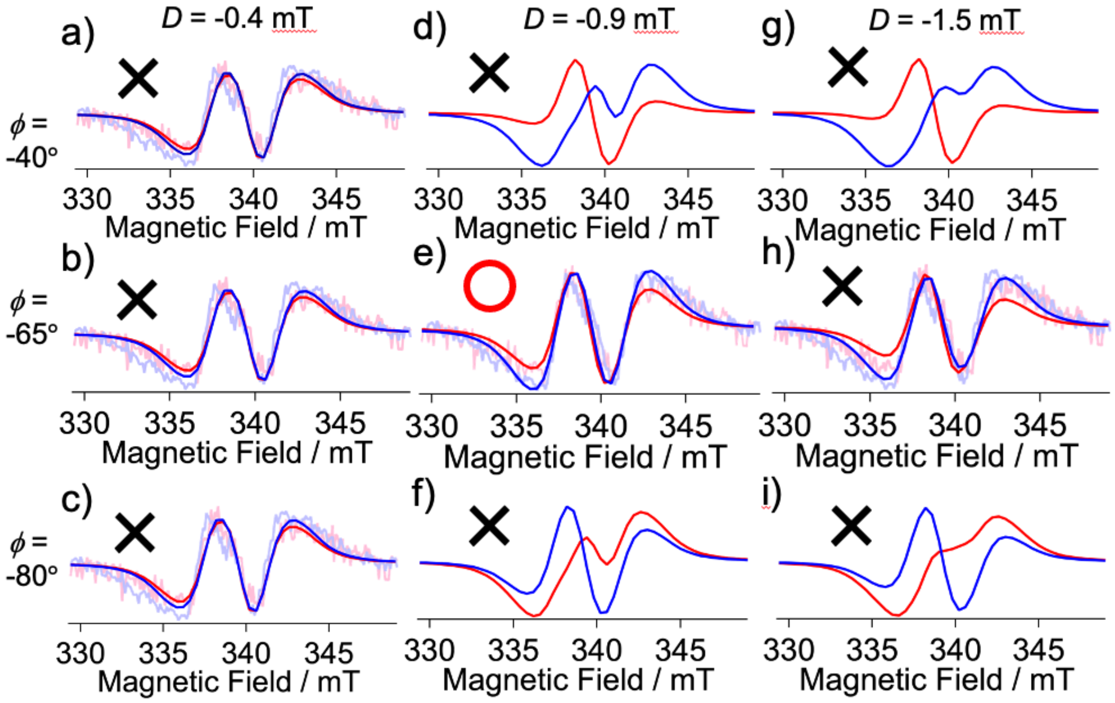

Supplementary Figure 4. Dependence of the spin-spin dipolar coupling parameters on the computed TREPR spectra for $\boldsymbol{B}_0$ // $\boldsymbol{L}$ (blue line corresponding to the dashed lines in Fig. 3d) and for $\boldsymbol{B}_0 \perp \boldsymbol{L}$ (red line corresponding to the solid lines in Fig. 3d). T_{2J}* = 3 ns corresponding to $1/(\pi T_{2J}^*)$ = 4 mT was fixed. T_{23}= 0.32 μs, J = 1.45 mT and θ = 58° were also applied. Firstly, T_{2J}* = 3 ns corresponding to $1/(\pi T_{2J}^*)$ = 4 mT was applied from the entire spectrum region of 18 mT (top arrows in Supplementary Figure 3) because the addition broadening effects by the spin-dipolar and the hyperfine couplings exist. In this regard, it is noted that the full of the hyperfine tensors (Supplementary Tables 2 and 3) are invoked from the literatures in the present analysis and are not the fitting parameters. At t = 0.2 μs, the fitting parameters are thus, T_{2J}*, D, J, T_{23} and the angles of θ and ϕ. First, we performed the spectrum computation of the CS state with using D = -0.9 mT with θ = 58 degrees and ϕ = -65 degrees, corresponding to the oxidation of W_B(H) by the photoinduced charge-separation in Fig. 3 at the x-ray geometry, as shown in e).

When a weak dipolar coupling constant of D = -0.4 mT was applied in a), b) and c), the spectrum differences were too small between $\boldsymbol{B}_0 \perp \boldsymbol{L}$ and $\boldsymbol{B}_0$ // $\boldsymbol{L}$ at any angle of ϕ and are deviated from the experimental results. For D = -0.9 mT (d, e, f) representing the separation distance of 1.45 nm between the radicals, the magnetophotoselection effects become prominent depending upon the position (ϕ) of the radical cation of W_B(H) in the X-Y-Z axis system of the FAD radical anion. The experimental results (light blue and light red lines) were reproduced with ϕ = -65 degrees (e). This is very consistent with the x-ray

conformation in Fig. 3a, indicating that the W_B(H) is oxidized by the charge-separation at 120 K. When assuming D = -1.5 mT from the separation distance of 1.2 nm between FAD and W_B(H) in Fig. 1, the experimental results again deviate from the calculations at any position of the oxidized tryptophan (g, h, i). We also computed the EPR spectra with D = -5.5 mT from the separation distance of 0.8 nm between FAD and W_A(H) together with θ = 85 degrees and ϕ = -80 degrees from Fig. 1. The computed spectra were highly deviated (not shown) from the experimental results, excluding the detection of the primary CS state composed of FAD^- and $W_A(H)^+$. Above results confirm that the TREPR spectra at 0.2 μs originate from the secondary CS state. From d), e), and f), the error in the angles of θ and ϕ are evaluated to be ±2 degrees. The error in the D coupling was evaluated to be ±0.37 mT in Supplementary Table 1.

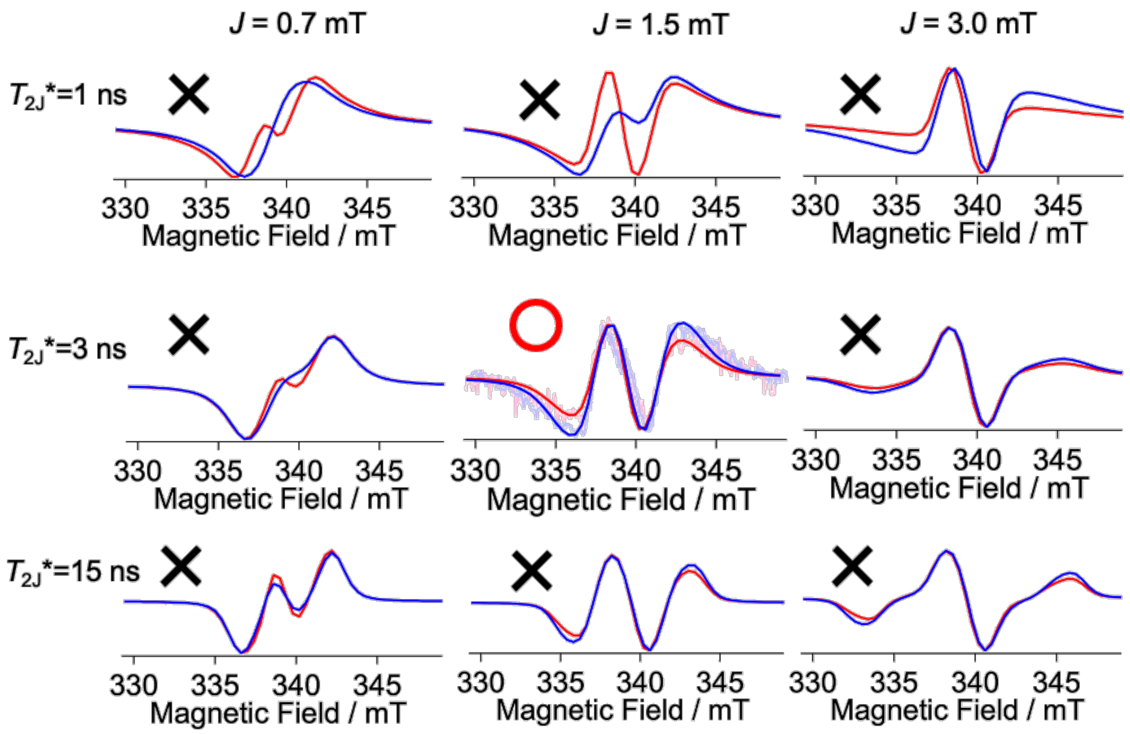

Supplementary Figure 5. Dependence of the exchange coupling parameters (J and T_{2J}*) on the computed TREPR spectra for $\boldsymbol{B}_0$ // $\boldsymbol{L}$ (blue) and for $\boldsymbol{B}_0 \perp \boldsymbol{L}$ (red). T_{23} = 0.32 μs, D = -0.90 mT, θ = 58°, and ϕ = −65° were fixed. To obtain the validities of the J parameter together with its distribution determined by T_{2J}*, we examined the susceptibility by the parameters of J and T_{2J}*. Because of these strong sensitivities to J and T_{2J}*, we are confident about the determinations of the several parameters on the molecular geometries and the isotropic exchange couplings from a series of the simulations. For the later delay times at 0.45 μs and 0.6 μs, the similar small but existing differences between the blue and red spectra caused by the laser polarization in Fig. 3 strongly denotes the geometries of the secondary CS states are unchanged by the delay time, while the J and T_{2J}* are time-dependent, as shown in Fig. 4. The error in J is evaluated to be ±0.1 mT.

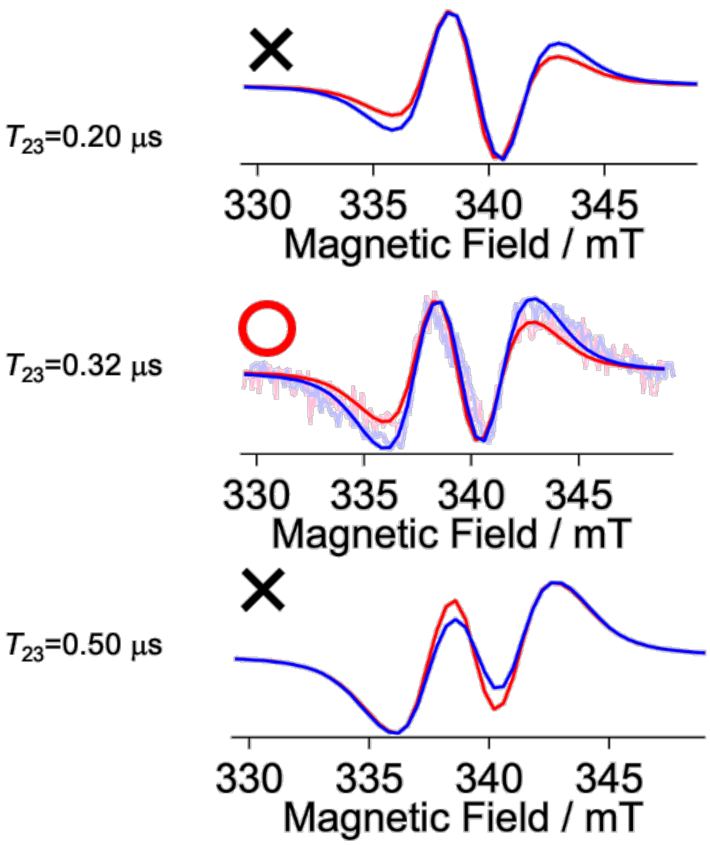

Supplementary Figure 6. Dependence of T_{23} value on the TREPR spectrum, showing strong susceptibility of this relaxation parameter. The inner A/E polarization becomes stronger than the outer A/E component when T_{23} is small. This effect can be used to characterize the fast solvation response in the W324F from *XlCry*-DASH at 120 K (Supplementary Figure 7). Notably, the calculated spectra obtained by T_{23} = 0.2 and 0.5 μs are evidently distinguished from the computed results in Supplementary Figures 4-5 using the other parameters. This denotes that the several input parameters of T_{23}, D, θ, ϕ, J, and T_{2J}* can be separately determined from the present simulation of the magnetophotoselection effects for the different delay times.

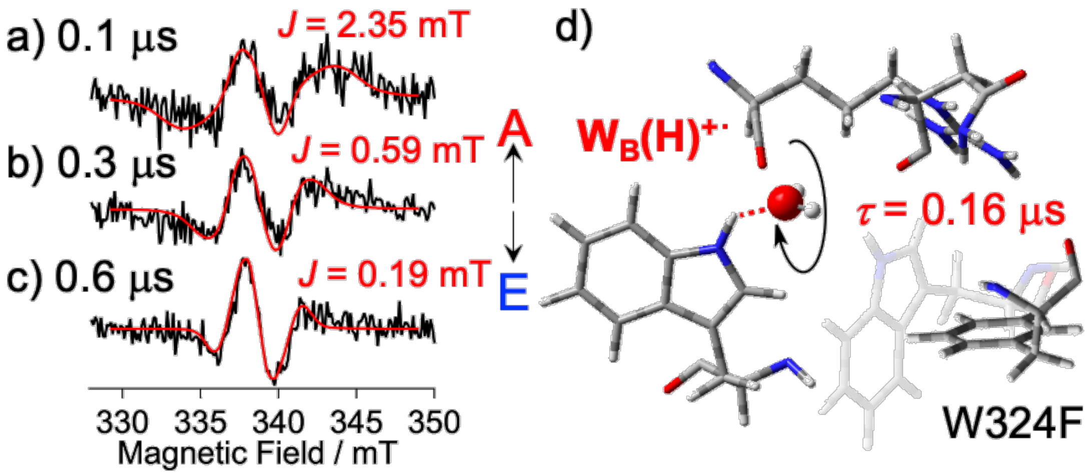

Supplementary Figure 7. a-c) t_d-dependent TREPR spectra obtained by the depolarized 450 nm laser irradiation of W324F from *XlCry*-DASH at 120 K, showing E/A/E/A patterns. The red lines were obtained with (J, T_{2J}*) = (2.35 mT, 4 ns), (0.59 mT, 5 ns) and (0.19 mT, 20 ns) at t_d = 0.1, 0.3 and 0.6 μs, respectively. While the E/A/E/A spin polarization pattern of the mutant is very similar to that of the WT (see Fig. 2), the width of the spectrum becomes quickly narrows within 0.3 μs. This is interpreted by an immediate decrease in J from 2.35 to 0.29 mT. From our SCRP model calculations (Supplementary Table 4), $T_{23} \approx 0.1$ μs and $T_1 \approx 0.2$ μs were required and explain both the drop in J and the narrowing of the TREPR spectrum, thus denoting that the solvation time is 0.16 μs. c) Accelerated solvation model by lack of the steric hinderance in the water molecule to account for the quick X_p response of dotted line in Fig. 5a for W324F. The shaded residue represents the W_C(H) conformation of the wild type obtained from the x-ray structure.

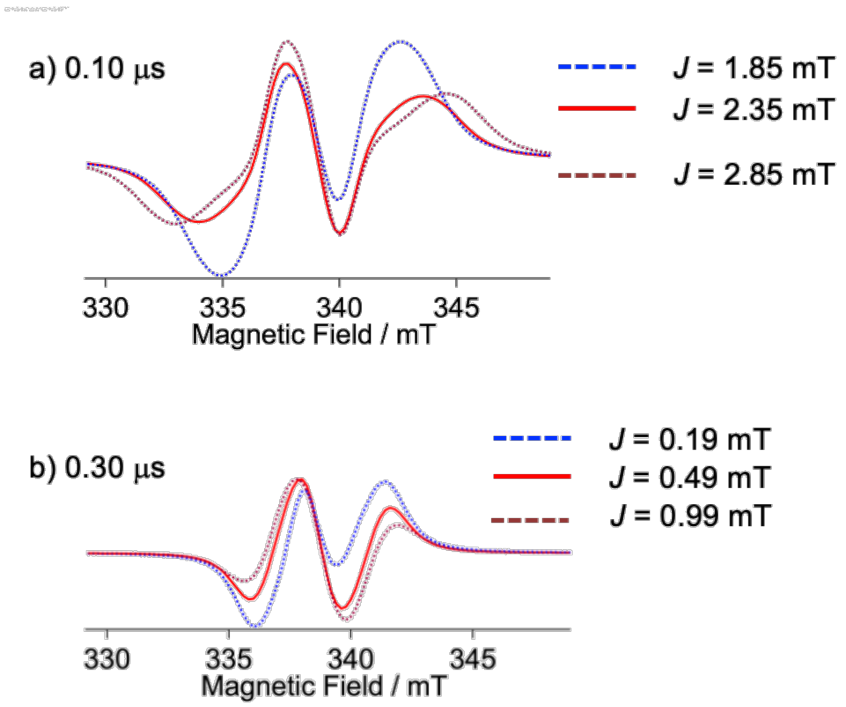

Supplementary Figure 8. Dependence of the exchange coupling parameters (*J*) on the computed TREPR spectra of W324F from *XlCry*-DASH at 120 K.

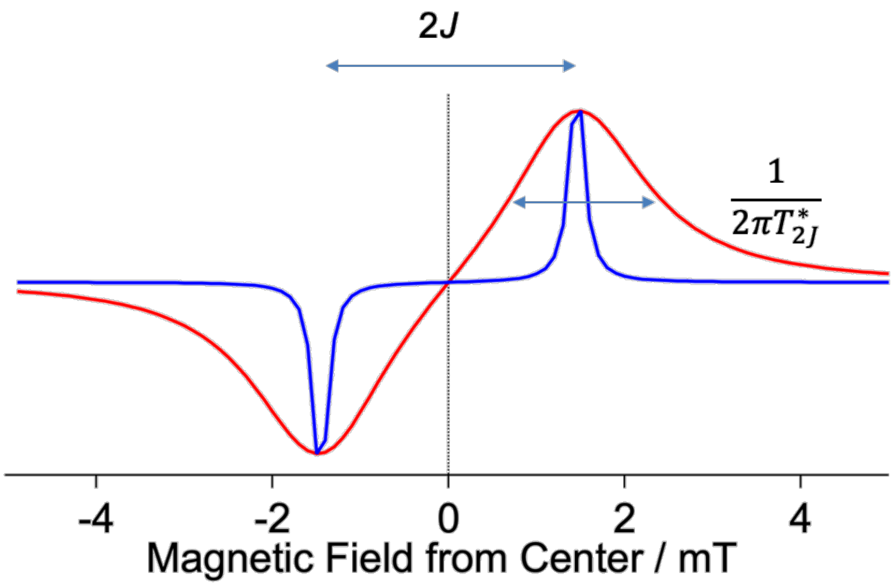

Supplementary Figure 9. Plots of the antiphase spectrum pattern determined by the Lorentzian line, as follows:

$$F(B_0) = \frac{1}{1 + T_{2J}^{*2}\left(\frac{g\beta B_0}{2} - J\right)^2} - \frac{1}{1 + T_{2J}^{*2}\left(\frac{g\beta B_0}{2} + J\right)^2}$$

(Supplementary Equation 1)

This was plotted as a function of the magnetic field (B_0) from EPR resonance center field with setting J = 1.45 mT determined at 0.2 μs (Fig. 3d). The red line was obtained with T_{2J}* = 3 ns, while T_{2J}* = 30 ns was used for the blue line. The peak-to-peak splitting (PPS) is unchanged by the variation in T_{2J}*, while the spectrum line-broadening is simply determined by 1/(2πT_{2J}*). This denotes that the red curve for B_0 > 0 is approximated to represent the inhomogeneous distribution function (i.e. the energetic disorder) in the J-coupling determined by the variation of 1/(2πT_{2J}*) = 1.9 mT for the secondary CS state. From this, the function $FF(B_0)$ were plotted against B_0 in Fig. 4a for B_0 > 0, as the distribution functions of the singlet-triplet gap i.e. PPS in Supplementary Figure 9, as follows:

$$FF(B_0) = \frac{1}{1 + T_{2J}^{*2}(g\beta B_0 - 2J)^2} - \frac{1}{1 + T_{2J}^{*2}(g\beta B_0 + 2J)^2}$$

(Supplementary Equation 2)

Relating to Supplementary Equation 2, from our previous report,[4] the microwave transition of ρ_{S+} affected by the exchange coupling was expressed by the stochastic-Liouville equation, as follows:

$$\begin{pmatrix}\rho_{S+}\\ \rho_{0+}\end{pmatrix} = \omega_1 \begin{pmatrix} -Q_+ - d + 2J + \omega_0 - \mathrm{i}\left(\frac{k_S + k_T}{2} + \frac{1}{T_{2J}^*}\right) & Q_- \\ Q_- & -Q_+ - 3d + \omega_0 - \mathrm{i}\left(k_T + \frac{1}{T_{2d}^*}\right) \end{pmatrix}^{-1} \begin{pmatrix}\rho_{S0}\\ \rho_{00} - \rho_{++}\end{pmatrix}$$

(Supplementary Equation 3)

where ρ_{S0} denotes the S-T_0 coherence developed by frequency of the energy difference between the |2> and |3> levels. Q_+ and Q_- are determined by the sum and difference in the Larmor frequencies of the two radicals in the SCRP, respectively. When the Q_- term is ignored as $2|J| >> Q_-$, the S-T_+ transition spectrum is described by the imaginary part of the ρ_{S+} under the very weak microwave strength (ω_1) with the angular frequency of ω_0. This spectrum corresponds to the first term of Supplementary Equation 2 when d, k_S and k_T terms are ignored. T_{2J}* thus causes a lifetime broadening as the uncertainty in the $2J$ value determined by the width $1/(\pi T_{2J}^*)$, as shown in Fig. 4a in the main text. Notably, the SCRP spectra were computed using Supplementary Equation 3 without ignoring the above ignored parameters to explain the experimental results.

Relevance between the width $1/(\pi T_{2J}^*)$ and the inhomogeneous distribution function Supplementary Equation 2 was checked below (Supplementary Figure 10).

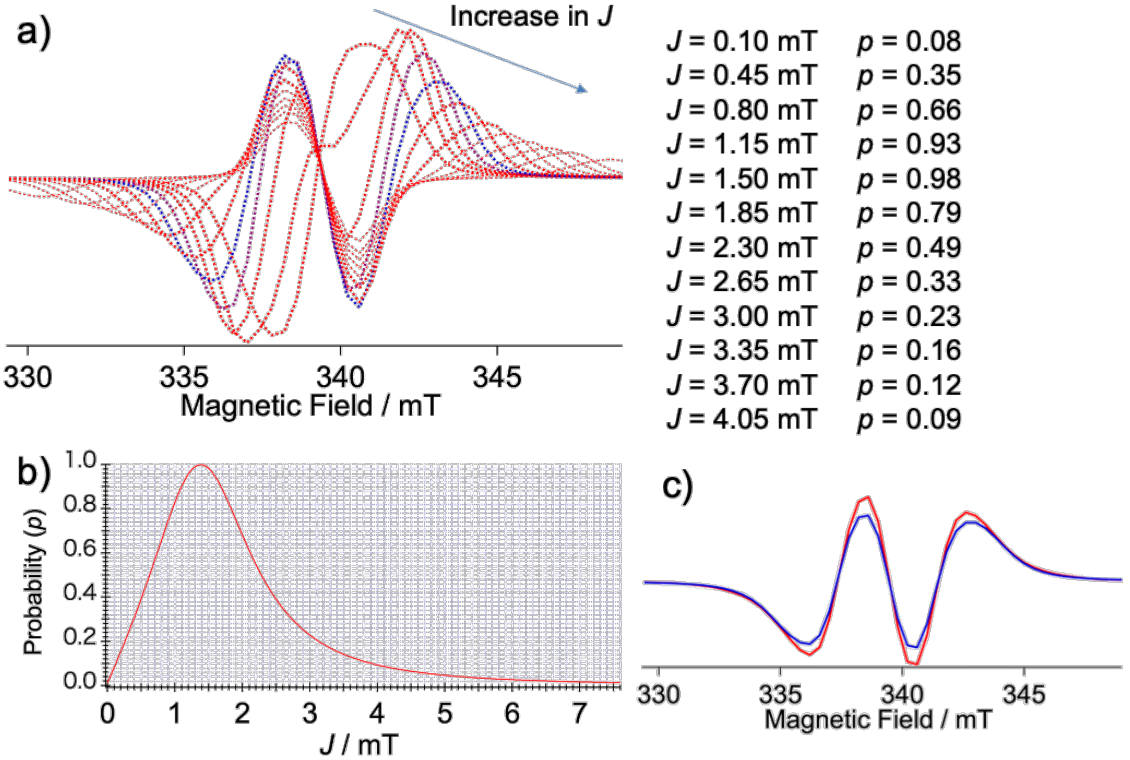

Supplementary Figure 10. a) SCRP spectra (transverse magnetization plotted as a function of B_0) computed with different J coupling values with T_{2J}^* = 15 ns for the depolarized laser excitations. Probabilities (p) of the exchange couplings were determined from the distribution function (Fig. 4a) in b). c): (Red) Averaged SCRP spectrum summed with weighting the p factors from the transverse magnetizations in a). (Blue) The SCRP spectrum obtained using T_{2J}^* = 4 ns and J = 1.45 mT.

To confirm whether the static distribution effects can be represented by the Lorentzian curve of Supplementary Equation 2 or not, we computed the transient magnetizations (i. e. the spin-polarized SCRP spectra) for the several different J parameters, as shown in a) with setting T_{2J}^* = 15 ns. For the different J values, we also obtained probabilities (p) of the J couplings in the protein environment from the distribution function by Supplementary Equation 2. The averaged EPR spectrum with weighting the p factor was calculated as shown by the red line in c). This was very consistent with the spectrum (blue line) computed with the single parameter of 1.45 mT with T_{2J}^* = 4 ns, denoting a very good compatibility representing the J-distribution by the present computation method originally reported in our previous studies.[4,5]

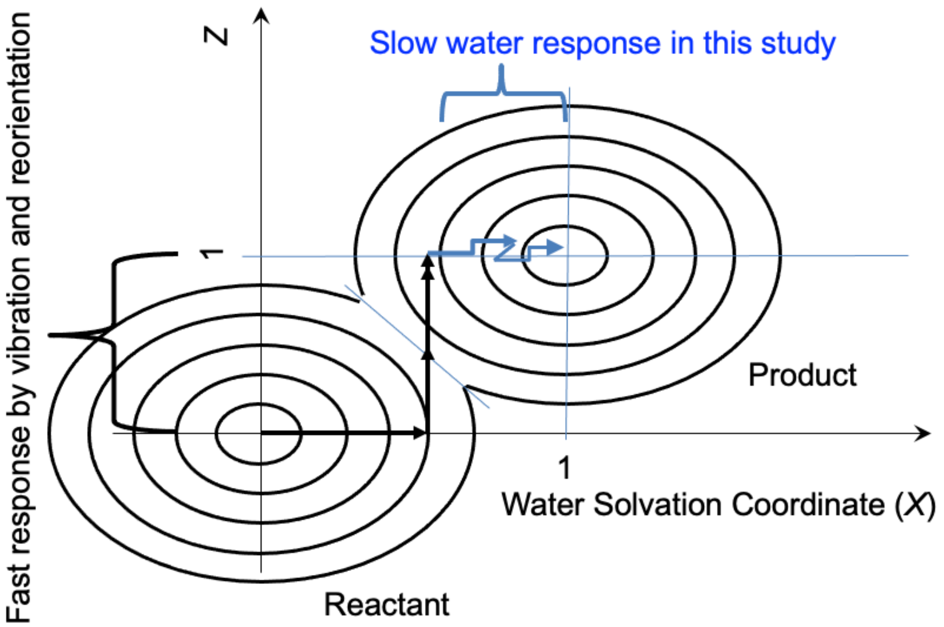

Supplementary Figure 11. The Sumi-Marcus electron-transfer model representing the fast nuclear response via vibration motions (vertical axis) and the slow response by the water reorientation (horizontal axis which is X in Fig. 4b) as the connection between the ET mechanism and the solvation dynamics in the present study to interpret the inhomogeneous distribution determined by T_{2J}*. The sub-microsecond response in Fig. 5a is regarded as the secondary relaxation in the product state by the slow reorientation at 120 K. In the ET reaction, several nuclear coordinates are participating including the energy relaxation process. The response time may vary with nature of the coordinate. In the present case, the secondary CS state, i.e. $FAD^{-\bullet}$ $W_B(H)^{+\bullet}$ could be generated at picoseconds regime via the vibrationally hot exciton in the primary CS state of $FAD^{-\bullet}$ $W_A(H)^{+\bullet}$. In this regard, the very quick nuclear and/or solvent motions may be involved forming the secondary CS immediately as reported previously.[6] Thus, the slow water dynamics in Fig. 4 is regarded as the secondary relaxation. In the present model of Fig. 4b, however, the reorganization energy was assumed to be determined by the single λ value (0.41 eV) for the simplicity of the treatment to predict the heterogeneous distribution of the exchange coupling (Fig. 4c).

Moreover, we approximated the distribution by the Gaussian functions (Fig. 4b) to simply evaluate the inhomogeneous J-distributions, although the non-relaxed states were treated. This is because a previous study apparently exhibited the Gaussian

distribution shapes to explain the solvation dynamics.[7] The present simplified assumption by the equilibrium Gaussian distribution is probably the reason for the slight differences between the distributions around the low-J regions in Fig. 4a and 4c because the distribution width is anticipated to be larger than the standard deviation of the equilibrium Gaussian distributions. Although more rigorous treatments invoking the Langevin equation should be forthcoming, which is out of scope in the present interpretations, there is no doubt about the present main conclusion on the involvements of the slow water reorientation causing the J-distribution.

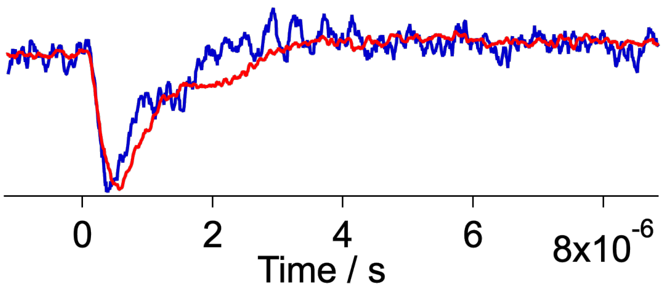

Supplementary Figure 12. Time profiles of the transverse magnetization of WT *XlCry*-DASH at 120 K with two different microwave powers (1 mW and 3 mW for the red log-lived and blue short-lived profiles, respectively at B_0 = 340 mT). Rabi oscillation frequency is 1.7 time higher in the blue profile than that in the red profiles and thus is caused by the transient nutation, demonstrating that the T_1 and T_2 relaxations (not the relaxation parameter determined by T_{2J}*) are larger than 1 microsecond. This wavy profile thus excludes the dynamic transverse relaxation with the nanosecond time regime and is consistent with a larger longitudinal spin relaxation times in Supplementary Table 1, denoting that the line shapes are determined by the inhomogeneous distribution of the exchange coupling.

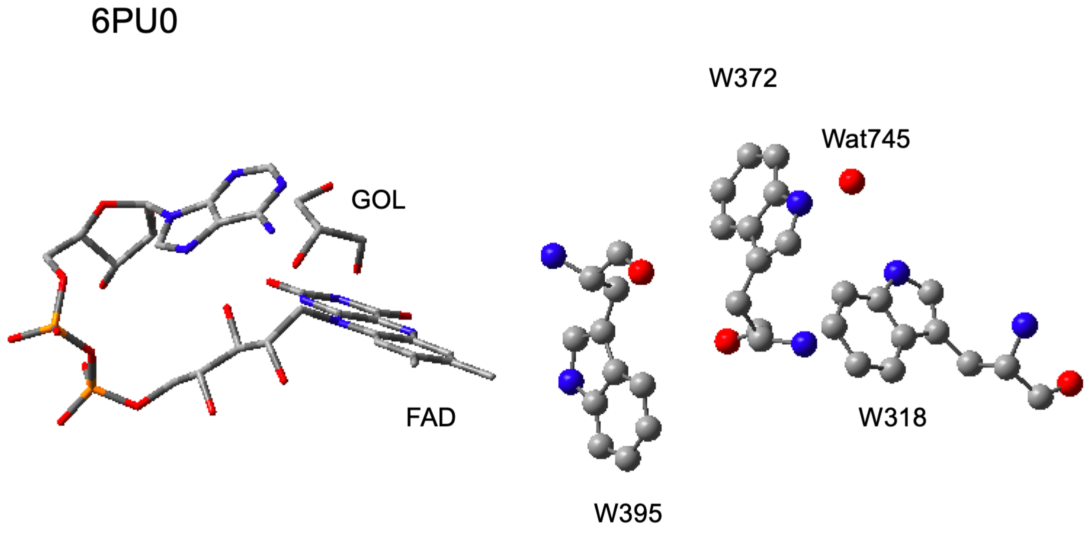

Supplementary Figure 13. Crystal structure of *Columba livia* CRY4 of pigeon from the PDB code: 6PU0. GOL represents glycerol and is not located between W372 and W318. The distance between GOL and Flavin is 0.93 nm, denoting that the solvation energy is negligibly small by GOL in the secondary RP state compared to the energy by the water solvation.

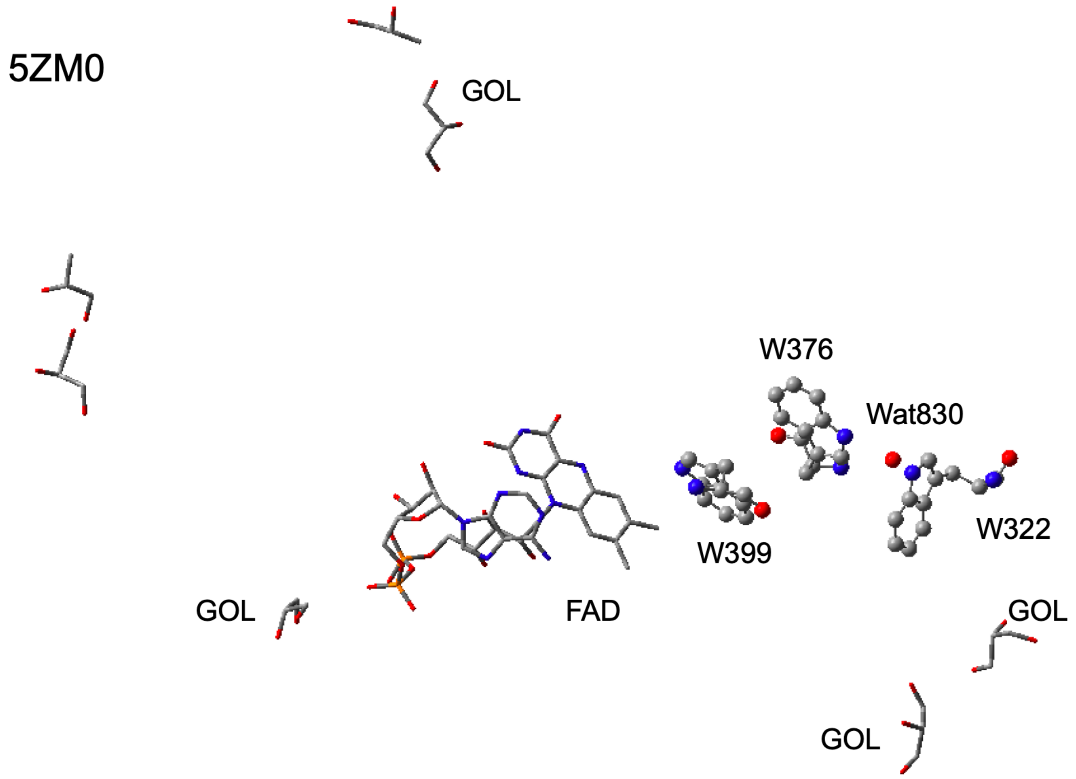

Supplementary Figure 14. Crystal structure of *Chlamydomonas reinhardtii* (PDB code:5ZM0). GOL represents glycerol and is not located between W376 and W322. Both distances between GOL molecules and flavin and between GOL and W376 are too long, denoting that the solvation energy contributions are negligible by GOL.

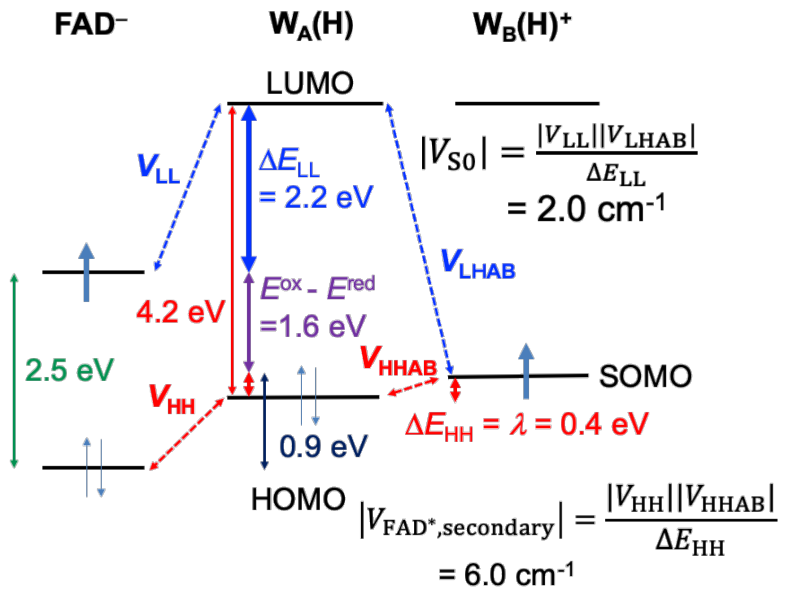

Supplementary Figure 15. Estimations and validities of the electronic couplings. Orbital energy levels in the secondary CS state of the molecules utilized for the electron couplings in protein. The dashed arrows represent the transfer integrals between the orbitals. Concerning the charge-recombination process generating the excited states (^{1}FAD* and ^{3}FAD*) in this figure, the hole-transfer tunneling scheme of FAD$^{-\bullet}\cdots$W$_A$(H)$\cdots$W$_B$(H)$^{+\bullet}$ → FAD$^{-\bullet}\cdots$W$_A$(H)$^{+\bullet}\cdots$W$_B$(H) → FAD*$\cdots$W$_A$(H)$\cdots$W$_B$(H) is applicable. In these cases, $|V_{S1}| = |V_{T1}| = |V_{FAD*}| = |V_{HHAB}||V_{HH}|/\Delta E_{HH}$ is obtained through the red dashed-line transfer integrals to estimate the electronic couplings ($|V_{S1}|$ and $|V_{T1}|$) mediated by W$_A$(H) in the secondary CS state. ΔE_{HH} is estimated to be the same as the reorganization energy (0.4 eV) for the degenerated hole transfer between the tryptophan residues. As for the recombination process leading to the ground state (G), the electron-transfer scheme of FAD$^{-\bullet}\cdots$W$_A$(H)$\cdots$W$_B$(H)$^{+\bullet}$ → FAD$\cdots$W$_A$(H)$^{-\bullet}\cdots$W$_B$(H) → FAD$\cdots$W$_B$(H)$\cdots$W$_C$(H) is applicable through the blue dashed arrows. Thus, $|V_{S0}| = |V_{LHAB}||V_{LL}|/\Delta E_{LL}$ is obtained where ΔE_{LL} denotes energy gap between the LUMOs in tryptophan and in FAD. ΔE_{LL} = 2.2 eV is estimated from the S$_0$ - S$_1$ excitation energies (2.5 eV and 4.2 eV in FAD and tryptophan, respectively), redox potentials ($E^{ox} - E^{red}$ = 1.6 eV) and ΔE_{HH} = 0.4 eV. ΔE_{LL} is thus five times larger than ΔE_{HH}, denoting that the tunneling barrier (blue vertical arrow) is significantly higher for the recombination to the ground state. This explains $|V_{S1}| \approx |V_{T1}| > |V_{S0}|$ in the main text. From $|V_{FAD*,secondary}| = |V_{HHAB}||V_{HH}|/\Delta E_{HH}$ with $|V_{HHAB}| = |V_{HH}|$, $|V_{HHAB}| = |V_{HH}|$ = 140 cm^{-1} was obtained. This coupling term accounts for the electron transfer rate of 6.4×10^{10} s^{-1} in the secondary charge-separation at the edge-to-edge separation (0.39 nm) between W$_A$(H) and W$_B$(H), as reported by I. A. Solov'yov.[8]

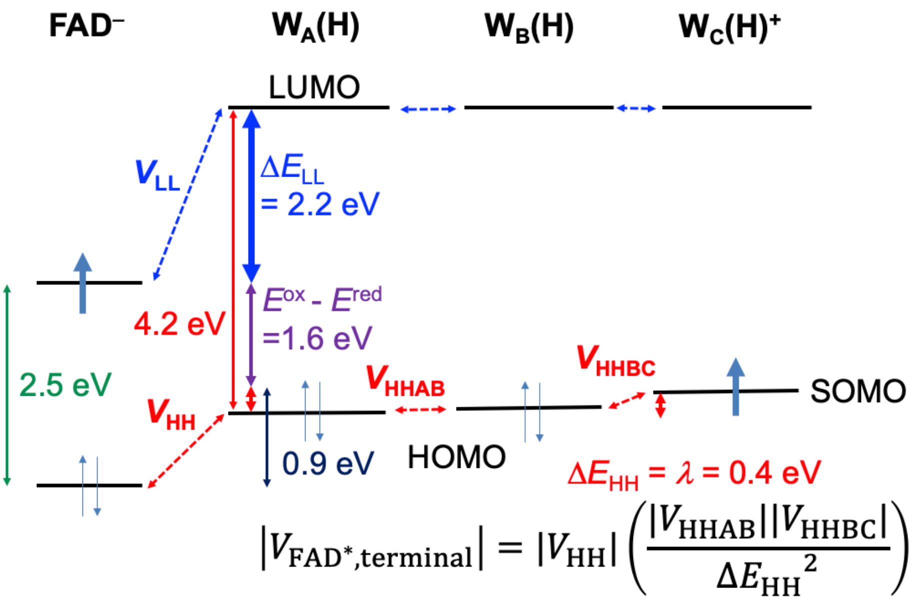

Supplementary Figure 16. Orbital energy levels in the terminal CS state of the FAD and tryptophan residues utilized for the electron tunneling in protein. The dashed arrows represent the transfer integrals between the orbitals. Concerning the charge-recombination to FAD*, the electronic coupling is represented as, $|V_{\mathrm{FAD*,\ terminal}}| = |V_{\mathrm{HHAB}}||V_{\mathrm{HHBC}}||V_{\mathrm{HH}}|/\Delta E_{\mathrm{HH}}^2$ by the seperexchange model. By using the superexchange model of $|V_{\mathrm{FAD*}}| = |V_{\mathrm{HHAB}}||V_{\mathrm{HH}}|/\Delta E_{\mathrm{HH}}$ in Supplementary Figure 15, $|V_{\mathrm{FAD*,\ terminal}}|/|V_{\mathrm{FAD*,secondary}}| = |V_{\mathrm{HHBC}}|/\Delta E_{\mathrm{HH}}$ is obtained for the electronic attenuation by the terminal CS.

The primary CS character can be participating in the secondary CS state as the wavefunction admixture via the electronic coupling. From the perturbation theory, this coefficient of the wavefunction participating to the secondary CS is readily evaluated to be $V_{\mathrm{HHAB}}/\ \lambda \approx (140\ \mathrm{cm}^{-1}/\ 3200\ \mathrm{cm}^{-1}) = 0.04$ from Supplementary Figure 15 that explains the time-dependence of the S-T gaps with their distributions (Fig. 4a). This denotes only 0.2 % of the primary CS character via the superexchange model around $X = 1$ in Fig. 4b, meaning that the primary CS character is much smaller than 1 % even at 0.2 microsecond because the solvation relaxation already proceeded with $X\mathrm{p} = 0.4$ in Fig. 4b. This well coincides with the magnetophotoselection results that showed the time-independent ***d***-direction of $(\theta,\ \phi) = (58°,\ -65°)$ with $\delta = 65°$ in Fig. 3a, as the dominant CS state.

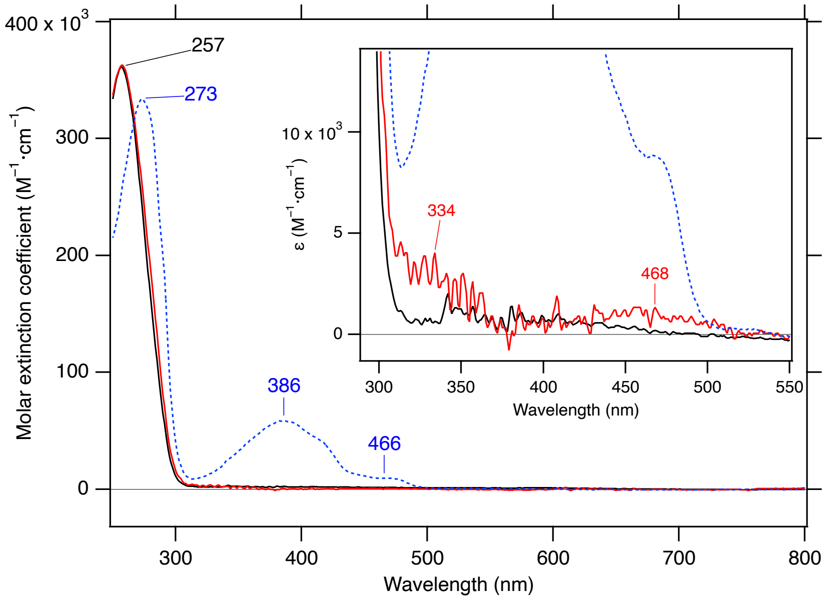

Supplementary Figure 17. UV-vis spectrum of *Xenopus laevis* CRY-DASH. Chromophore-bound protein with ferricyanide treatment (blue broken line), chromophore-removed protein without (black solid line) and with (red solid line) ferricyanide treatment proteins. Molar extension coefficient of chromophore-removed CRY-DASH was estimated by protein concentration by Bradfrod method, the molecular mass of the calculated His_6-tagged protein, and the absorption spectrum. The absorption spectrum of chromophore-bound protein was superimposed with FAD cofactor's extinction coefficient as 1.0×10^4 M^{-1} cm^{-1} at 450 nm.

Oxindole absorption peak is reported to appear around 340 nm with an extinction coefficient of 1×10^4 M^{-1} cm^{-1}.[9] Oxidized tyrosine species from DOPA exhibits similar absorption bands around 300 nm and 470 nm.[10] Estimated from the enlarged absorbances around 330 nm (Inset), oxidized forms of tryptophan or tyrosine are thus 0.3 residues/molecule. A small absorption around 470 nm suggests existence of oxidized tyrosine of dopachrome with the extinction coefficient of 3.7×10^3 M^{-1} cm^{-1} at 475 nm,[11,12] indicating that 0.1 residues/molecule are oxidized in tyrosine. Possible candidates for oxidized tryptophan and tyrosine residues were shown in Supplementary Figure 18, where 8 tryptophan and 12 tyrosine residues were exposed to the surface of the protein including W324 corresponding to W_C(H). (See below.) It is thus concluded that the oxidation of W_C(H) occurs less than 0.02 residues per molecule from a very small amount of free iron impurity. This minor (< 2 %) possibility of pre-oxidation in W_C(H) is very consistent to the observation of the terminal CS state assigned to $FAD^{-\bullet}\cdots W_A(H)\cdots W_B(H)\cdots W_C(H)^{+\bullet}$ in Supplementary Figure 1 at the elevated temperature. This strongly supports the significant inhibition of the terminal CS event

causing the water reorientation to $W_B(H)^{+\bullet}$ at the secondary CS state at 120 K.

(a)

```
Xl_CRY_DASH                 MCVPSRVIICLLRNDLRLHDNEVLHWAHRNADQIVPLYCFDPRHYVGTHY   50
Synechocystis_CRY-DASH      MKHVPPTVLVWFRNDLRLHDHEPLHRALKSGLAITAVYCYDPRQFAQTHQ   50
                            *   . .::  :********:* ** * :..  *..:**:***::. **

Xl_CRY_DASH                 FNFPKTGPHRLKFLLESVRDLRITLKKKGSNLLLRRGKPEEVIEDLVKQL  100
Synechocystis_CRY-DASH      G-FAKTGPWRSNFLQQSVQNLAESLQKVGNKLLVTTGLPEQVIPQIAKQI   99
                            *.**** * :** :**::*  :*:* *.:**:  * **:** ::.**:

Xl_CRY_DASH                 GNVSAVTLHEEATKEETDVESAVKQACTRLGIKYQTFWGSTLYHREDLPF  150
Synechocystis_CRY-DASH      N-AKTIYYHREVTQEELDVERNLVKQLTILGIEAKGYWGSTLCHPEDLPF  148
                            . ..::  *.*.*:** ***  : :  * ***: : :***** * *****

Xl_CRY_DASH                 RHISSLPDVYTQFRKAVET-QGKVRPTFQMPDKLKPLPSGLEEGSVPSHE  199
Synechocystis_CRY-DASH      S-IQDLPDLFTKFRKDIEKKKISIRPCFFAPSQLLPSPNIKLELTAPPPE  197
                            *..***::*:*** :*. : .:** *  *.:* * *.   * :.*. *

Xl_CRY_DASH                 DFDQQDPLTDPRTAFPCSGGESQALQRLEHYFWETNLVASYKDTRNGLIG  249
Synechocystis_CRY-DASH      FFPQIN--FDHRSVLAFQGGETAGLARLQDYFWHGDRLKDYKETRNGMVG  245
                            * * :   * *:.:. .***: .* **:.***. : : .**:****::*

Xl_CRY_DASH                 LDYSTKFAPWLALGCVSPRYIYEQIGKYEKERTANQSTYWVIFELLWRDY  299
Synechocystis_CRY-DASH      ADYSSKFSPWLALGCLSPRFIYQEVKRYEQERVSNDSTHWLIFELLWRDF  295
                            ***:**:*******:***:**::: :**:**.:*:**:*:********:

Xl_CRY_DASH                 FRFVALKYGRRIFFLRGLQDKDIPWKRDPKLFDAWKEGRTGVPFVDANMR  349
Synechocystis_CRY-DASH      FRFVAQKYGNKLFNRGGLLNKNFPWQEDQVRFELWRSGQTGYPLVDANMR  345
                            ***** ***.::*   ** :*::**:.*   *: *:.*:** *:******

Xl_CRY_DASH                 ELAMTGFMSNRGRQNVASFLTKDLGIDWRMGAEWFEYLLVDYDVCSNYGN  399
Synechocystis_CRY-DASH      ELNLTGFMSNRGRQNVASFLCKNLGIDWRWGAEWFESCLIDYDVCSNWGN  395
                            ** :**************** *:****** ******  *:*******:**

Xl_CRY_DASH                 WLYSAGIGNDPRENRKFNMIKQGLDYDSGGDYIRLWVPELQQIKGGDAHT  449
Synechocystis_CRY-DASH      WNYTAGIGNDARDFRYFNIPKQSQQYDPQGTYLRHWLPELKNLPGDKIHQ  445
                            * *:******.*: * **: **. :**. * *:* *:***::: *.. *

Xl_CRY_DASH                 PWALSNASLAHANLSLGETYPYPIVMAPEWSRHINQKPAGSWEKSARRGK  499
Synechocystis_CRY-DASH      PWLLSATEQKQWGVQLGVDYPRPCVNFHQSVEARRKIEQMGVIA------  489
                            ** ** :.  : .:.**  ** * *    :   .  .:     .

Xl_CRY_DASH                 GPSHTPKQHKNRGIDFYFSRNKDV                             523
Synechocystis_CRY-DASH      ------------------------                             489
```

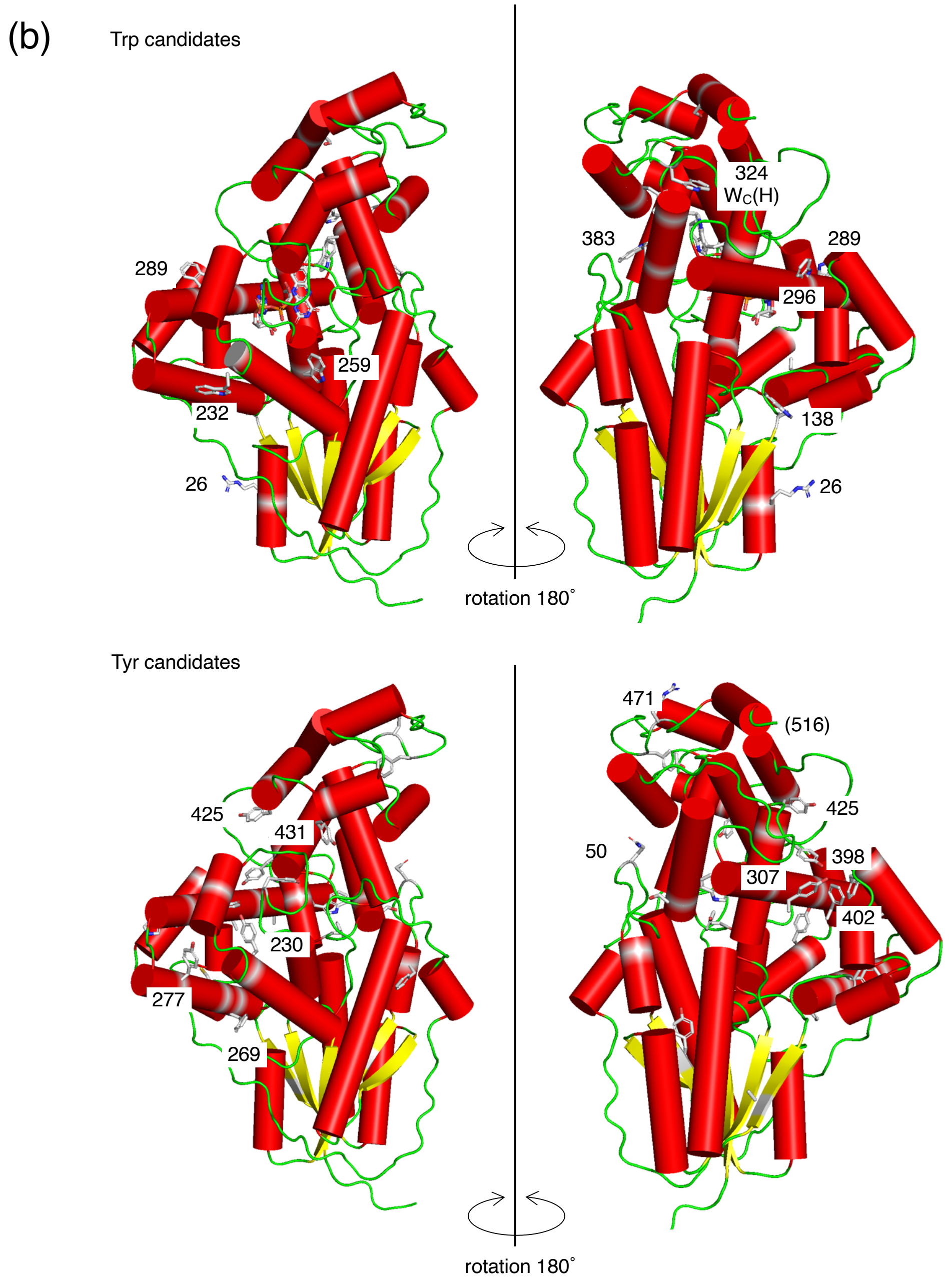

Supplementary Figure 18. Exploring candidates for oxidized tryptophan and tyrosine residues. (a) Comparison of the amino acid sequences of *Xenopus laevis* and *Synechocystis* CRY-DASHs. The amino acid residues corresponding to tryptophan and tyrosine in *Xenopus laevis* CRY-DASH are colored by yellow and green, respectively. (b) For the crystal structure of *Synechocystis* CRY-DASH (PDB ID: 1NP7),[13] the residues corresponding to tryptophans (top) and tyrosines (bottom) in *Xenopus laevis* CRY-DASH are shown as sticks. The residues whose side chains are considered to be exposed on the surface are indicated by amino acid numbers of *Xenopus laevis* CRY-

DASH.

Supplementary Tables

Supplementary Table 1. EPR parameters for the simulations of the t_d-dependent EPR data (Fig. 3d) of the photoinduced CS states at 120 K.

Delay times (t_d) / μs	D / mT [a)]	J / mT	T_{2d}*/ ns	T_{2J}*/ ns	T_1 / μs	T_{23} / μs	k_S / 10^5 s^{-1}	k_T / 10^4 s^{-1}
0.20	-0.90 (±0.37)	1.45	10	3.0	4	0.32	2.5	5.0
0.45	-0.90 (±0.25)	0.55	15	15	4	0.22	2.5	5.0
0.60	-0.90 (±0.15)	0.40	25	20	4	0.28	2.5	5.0

a) Errors in parentheses were estimated from the line-widths determined by $1/(2\pi T_{2d}*)$ originating from heterogeneities of 3d in the T_0-$T_\pm$ transition energies.[5] Distributional deviation in the center-to-center distance (r_{CC}) is estimated to be ±0.08 nm form the variation in the D value of ±0.15 mT at t_d = 0.60 μs.

Supplementary Table 2. The hyperfine-tensor components (mT) utilized for the tryptophan radical cation for the computations of the TREPR spectra.

	$TrpH^{•+}$		
Atom positions	A_{XX}	A_{YY}	A_{ZZ}
$H\beta_1$ [a)]	1.39	1.39	1.39
$H\beta_2$ [a)]	1.39	1.39	1.39
$^{14}N_1$ [b)]	0.14	0.14	0.88
H_2 [b)]	−0.11	−0.49	−0.37
H_5 [b)]	−0.22	−0.94	−0.72
H_7 [b)]	−0.17	−0.72	−0.55
H_1 [b)]	−0.17	−0.72	−0.55

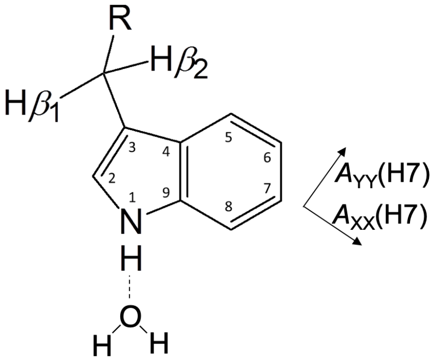

a) From the coupling constants of the beta protons, spin density (ρ_{C3}) at the C3 position is estimated to be ρ_{C3} = 0.50 using $a_{H\beta}{}^{iso} = \rho_{C3}\, Q_b \cos^2\xi$ representing the hyperconjugation effect on the hyperfine coupling constants with Q_b = 6 mT and $\cos^2\xi$ = 0.43. This is roughly consistent with ρ_{C3} = 0.44 reported for a complex of water and tryptophan cation radical.[14] However, $a_{H\beta}{}^{iso}$ = 1.39 mT would include the line-broadening effect by the hyperfine couplings from H6 and H8 protons and by the superhyperfine by the water protons, those of which were ignored in this table due to the minor spin densities at C6, C8 and O positions. For the ternary CS state in Supplementary Figure 1, $a_{H\beta}{}^{iso}$ = 1.29 mT was applied corresponding to ρ_{C3} = 0.46 reported for a complex of water and tryptophan cation radical.[14]

b) Estimated from the reported coupling constants of the neutral radical and the computed spin densities (ρ_{N1} = 0.16, ρ_{C2} = 0.13, ρ_{C5} = 0.26, and ρ_{C7} = 0.17) at the aromatic ring for the complex of water and tryptophan cation radical.[14] Because of $\rho_{N1} \approx \rho_{C7}$, the principal values in H1 proton were assume to be the same as the values for H7. An example of the X and Y principal axes, these axes directions are represented in H7. The Z axes are perpendicular to the aromatic plane for all of the anisotropic couplings.

Supplementary Table 3. The hyperfine-tensor components (mT) utilized for the FAD radical anion for the computations of the TREPR spectra.

	FAD•−		
Atom positions	A_{XX}	A_{YY}	A_{ZZ}
$^{14}N_{10}$ [a)]	0.0	0.0	0.89
$^{14}N_{5}$ [a)]	0.0	0.0	1.89
H8(-CH_3)	0.35 [b)]	0.35 [b)]	0.35 [b)]
H6	0.35 [b)]	0.35 [b)]	0.35 [b)]

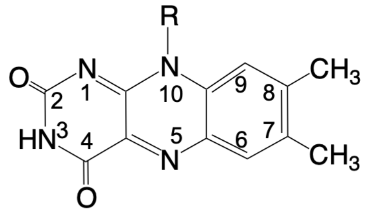

a) Taken from the EPR study by Okafuji *et al.*.[2] The A_{ZZ} principal axes are perpendicular to the aromatic plane.
b) Obtained by an averaged value determined by $[3A_{iso}(H8) + A_{iso}(H6)]/4$ where $A_{iso}(H8)$ = 10.45 MHz in -CH_3 and $A_{iso}(H6)$ = 8.2 MHz because these four isotropic coupling constants from these protons are similar.[2] We expect that the -CH_3 group freely rotate at 120 K. Thus, only the isotropic couplings were considered at 120 K and at 240 K.

Supplemental Table 4. Parameters for the simulations of the time-dependent EPR data (Supplementary Figure 7a-c) of the photoinduced CS states of W324F at 120 K.

Delay times (t_d) / μs	D / mT	J / mT	T_{2d}*/ ns	T_{2J}*/ ns	T_1 / μs	T_{23} / μs	k_S / 10^5 s^{-1}	k_T / 10^4 s^{-1}
0.10	-0.90 (±0.25)	2.35	15	4.0	0.10	0.10	2.5	5.0
0.30	-0.90 (±0.15)	0.59	15	5.0	0.25	0.07	2.5	5.0
0.60	-0.90 (±0.15)	0.19	15	20	0.50	0.07	2.5	5.0

Supplemental Table 5. Parameters for the simulations of the TREPR data (red lines in Supplementary Figure 1) of the photoinduced CS state $FAD^{-\bullet}\cdots W_C(H)^{+\bullet}$ of WT protein at 240 K.

D / mT[a)]	J / μT [a)]	T_{2d}*/ ns	T_{2J}*/ ns	T_1 / μs	T_{23} / μs	k_S / 10^5 s^{-1}	k_T / 10^5 s^{-1}
-0.40 (±0.15)	22	23	20	1.4	1.8	1.7	1.0

a) r_{CC} = 1.91 nm (±0.08 nm) is estimated from the D value using $D(r_{CC})$ / mT = −2.78 / $(r_{CC}/\text{nm})^3$. From $2J$ = 44 μT, $|V_{S1,\text{teminal}}|$ = 1.6 cm^{-1}, $|V_{T1,\text{teminal}}|$ = 1.5 cm^{-1} and $|V_{S0,\text{teminal}}|$ = 0.5 cm^{-1} were obtained as the electronic couplings of the terminal CS state using Equation 1 and the potentials at X = 1 in Fig. 4b at 240 K.

Supplementary Notes

Supplementary Note 1. Contribution of the anisotropic spin-spin exchange coupling:

It is reported that a perturbation from the excited state may allow the spin-orbit coupling (SOC) to participate in the exchange coupling, resulting in the anisotropy in the exchange parameter, as the origin of the g-factor is in organic radical species in frozen systems. (Bencini and Gatteschi, "EPR of Exchange Coupled Spins" (2012) Dover Publication Inc. pp. 27.)[15] Based upon their formalism, this anisotropic exchange interaction is negligibly minor in the present CS state because 1) third order perturbation treatment of the SOC via the excited state is required and 2) the excited state energy in FAD^{-}•* possessing the n-orbital character must be very high, which is relevant to very small g-anisotropy in Supplementary Figure 2.

Furthermore, from our present analyses of the electron spin polarization, it was demonstrated that the time-dependent J is caused by isotropic transfer integrals of V between the separated FAD^{-}• and $W_B(H)^{+}$• radicals with Equation 1. As described in the manuscript, the transfer integrals are simply determined by the orbital overlap of V_{HH} between FAD and W_A(H) and by V_{HHAB} between W_B(H) and W_A(H) via the bridge-mediated tunneling interactions. These orbital overlaps are all isotropic interactions with $|V_{HHAB}|$ = 140 cm^{-1} (Supplementary Figure 15). Because this coupling term accounts for the electron transfer rate of 6.4×10^{10} s^{-1} at the contact edge-to-edge separation (0.39 nm) between W_A(H) and W_B(H) (Supplementary Figure 15), it is concluded that the S-T gaps in Fig. 4 are all isotropic in the present system. This means that the J-coupling in the present distant radical pair is not anisotropic and is dominated by the isotropic transfer integrals as the configuration interaction. This is self-consistent with the long-range electronic coupling causing the distant exchange interaction isotopically at 1.4 nm for the secondary CS state. Overall, the anisotropic exchange coupling is concluded to be negligible in the present study.

Supplementary Note 2. Possibility of glycerol role on the solvent dynamics and the electron tunneling:

When the glycerol binding is taken instead of the water between W_B(H) and W_C(H), the electron tunneling matrix element in Supplementary Table 1 must be weakened through increased number of the sigma bonds (3 bonds) from glycerol in the tunneling route via W_BH ··· (H)O-CH_3-CH_2(CH_2OH)-O(H) ··· W_CH, as an example, while W_BH···(H)O(H)···W_CH do not possess the sigma bonds in the case of the water binding. From the Pathways model with per-unit penalty of ε_C = 0.6 per the aliphatic bond, the attenuation from the secondary CS to the terminal CS is obtained to be ε = 0.0025 instead of 0.07 in Table 1. This is highly deviated from the experimental result of 0.08. Furthermore, three of the crystal structures of the PDB codes 6X24, 5ZM0 (Supplementary Figure 14) and 6PU0 (*Columba livia* CRY4 of pigeon: Supplementary Figure 13) are reported on cryptochromes to which the glycerol molecules are bound. Like Fig. 1, the CRY4 was also found to possess the relevant water binding site between W_B(H) and W_A(H). However, glycerol molecules are not bound to these positions but the different sites for three of the cryptochromes as shown in Supplementary Figure 14. Because it is widely accepted that glycerol molecules play a role to surround the protein surface area to protect the native protein structure, as reported by Vagenende et al.,[16] we exclude the possibility of the glycerol binding to impact the solvation dynamics in Fig. 4.

Supplementary References

1 Kay, C. W. M., Bittl, R., Bacher, A., Richter, G. & Weber, S. Unambiguous Determination of the g-Matrix Orientation in a Neutral Flavin Radical by Pulsed Electron−Nuclear Double Resonance at 94 GHz. *J. Am. Chem. Soc.* **127**, 10780-10781, (2005).

2 Okafuji, A., Schnegg, A., Schleicher, E., Möbius, K. & Weber, S. G-Tensors of the Flavin Adenine Dinucleotide Radicals in Glucose Oxidase: A Comparative Multifrequency Electron Paramagnetic Resonance and Electron − Nuclear Double Resonance Study. *J. Phys. Chem. B* **112**, 3568-3574, (2008).

3 Bleifuss, G. *et al.* Tryptophan and Tyrosine Radicals in Ribonucleotide Reductase: A Comparative High-Field EPR Study at 94 GHz. *Biochemistry* **40**, 15362-15368, (2001).

4 Hasegawa, M. *et al.* Regulated Electron Tunneling of Photoinduced Primary Charge-Separated State in the Photosystem II Reaction Center. *J. Phys. Chem. Lett.* **8**, 1179-1184, (2017).

5 Miura, T. *et al.* Geometries, Electronic Couplings, and Hole Dissociation Dynamics of Photoinduced Electron–Hole Pairs in Polyhexylthiophene–Fullerene Dyads Rigidly Linked by Oligophenylenes. *J. Am. Chem. Soc.* **138**, 5879-5885, (2016).

6 Lacombat, F. *et al.* Ultrafast Oxidation of a Tyrosine by Proton-Coupled Electron Transfer Promotes Light Activation of an Animal-like Cryptochrome. *J. Am. Chem. Soc.* **141**, 13394-13409, (2019).

7 Tominaga, K., Walker, G. C., Jarzeba, W. & Barbara, P. F. Ultrafast Charge Separation in Adma - Experiment, Simulation, and Theoretical Issues. *J. Phys. Chem.* **95**, 10475-10485, (1991).

8 Sjulstok, E., Olsen, J. M. H. & Solov'yov, I. A. Quantifying electron transfer reactions in biological systems: what interactions play the major role? *Sci Rep-Uk* **5**, (2015).

9 Edeson, S. J. *et al.* Studies on the stereochemical assignment of 3-acylidene 2-oxindoles. *Org Biomol Chem* **12**, 3201-3210, (2014).

10 Burzio, L. A. & Waite, J. H. Reactivity of peptidyl-tyrosine to hydroxylation and cross-linking. *Protein Sci.* **10**, 735-740, (2001).

11 Graham, D. G. & Jeffs, P. W. Role of 2,4,5-Trihydroxyphenylalanine in Melanin Biosynthesis. *J. Biol. Chem.* **252**, 5729-5734, (1977).

12 Behbahani, I., Miller, S. A. & Okeeffe, D. H. A Comparison of Mushroom Tyrosinase Dopaquinone and Dopachrome Assays Using Diode-Array Spectrophotometry - Dopachrome Formation Vs Ascorbate-Linked Dopaquinone Reduction. *Microchem. J.* **47**, 251-260, (1993).

13 Brudler, R. *et al.* Identification of a new cryptochrome class: Structure, function, and evolution. *Mol Cell* **11**, 59-67, (2003).

14 Pogni, R. *et al.* A tryptophan neutral radical in the oxidized state of versatile peroxidase from Pleurotus eryngii - A combined multifrequency EPR and density functional theory study. *J. Biol. Chem.* **281**, 9517-9526, (2006).

15 Bencini, A. & Gatteschi, D. *EPR of Exchange Coupled Systems*. (Dover Publications, Inc., 2012).

16 Vagenende, V., Yap, M. G. S. & Trout, B. L. Mechanisms of Protein Stabilization and Prevention of Protein Aggregation by Glycerol. *Biochemistry* **48**, 11084-11096, (2009).
